# The genetic spectrum of familial hypercholesterolemia in south-eastern Poland

**DOI:** 10.1016/j.metabol.2015.10.018

**Published:** 2016-03

**Authors:** Mahtab Sharifi, Małgorzata Walus-Miarka, Barbara Idzior-Waluś, Maciej T. Malecki, Marek Sanak, Ros Whittall, Ka Wah Li, Marta Futema, Steve E. Humphries

**Affiliations:** a Centre for Cardiovascular Genetics, Institute of Cardiovascular Sciences, University College London, London, UK; b Department of Metabolic Diseases, Jagiellonian University Medical School, Kraków, Poland; c Department of Medical Didactics, Jagiellonian University Medical School, Kraków, Poland; d 2nd Department of Internal Medicine, Institute of Molecular Biology and Clinical Genetics, Jagiellonian University Medical School, Kraków, Poland

**Keywords:** FH, familial hypercholesterolemia, SNP, single nucleotide polymorphism, *PCSK9*, protein convertase subtilisin/kexin 9 gene, *APOB*, apolipoprotein B gene, Familial hypercholesterolemia, *LDLR* mutation, LDL-C gene score

## Abstract

**Background:**

Familial hypercholesterolemia (FH) is a common autosomal dominant disorder with a frequency of 1 in 200 to 500 in most European populations. Mutations in *LDLR*, *APOB* and *PCSK9* genes are known to cause FH. In this study, we analyzed the genetic spectrum of the disease in the understudied Polish population.

**Materials and methods:**

161 unrelated subjects with a clinical diagnosis of FH from the south-eastern region of Poland were recruited. High resolution melt and direct sequencing of PCR products were used to screen 18 exons of *LDLR*, a region of exon 26 in the *APOB* gene and exon 7 of *PCSK9*. Multiplex ligation-dependent probe amplification (MLPA) was performed to detect gross deletions and insertions in *LDLR*. Genotypes of six LDL-C raising SNPs were used for a polygenic gene score calculation.

**Results:**

We found 39 different pathogenic mutations in the *LDLR* gene with 10 of them being novel. 13 (8%) individuals carried the p.Arg3527Gln mutation in *APOB*, and overall the detection rate was 43.4%. Of the patients where no mutation could be found, 53 (84.1%) had a gene score in the top three quartiles of the healthy comparison group suggesting that they have a polygenic cause for their high cholesterol.

**Conclusions:**

These results confirm the genetic heterogeneity of FH in Poland, which should be considered when designing a diagnostic strategy in the country. As in the UK, in the majority of patients where no mutation can be found, there is likely to be a polygenic cause of their high cholesterol level.

## 1 Introduction

Familial hypercholesterolemia (FH) is an autosomal dominant disorder with a frequency of 1 in 200 to 500 in European populations [1]. It is characterized by a raised concentration of low-density lipoprotein cholesterol (LDL-C) and high risk of premature coronary heart disease [2].

Mutations in the *LDLR* gene, the *APOB* gene and gain-of-function mutations in the *PCSK9* gene are known to cause FH [3]. Usually an FH-causing mutation can be found in 60–80% of patients with a clinical diagnosis of definite FH and 20–30% of those with possible FH [4]. In those where no causative mutation is found, there is a strong possibility that there may be a polygenic cause for FH [5].

In Poland, FH is an under-diagnosed condition with only 20% of the cases estimated to be diagnosed to date [6]. The aim of this study is to assess the spectrum of FH-causing mutations in the Malopolska population in east-southern Poland.

## 2 Methods

### 2.1 Subjects

161 unrelated Caucasians patients with a clinical diagnosis of FH based on Simon Broome criteria [7] were recruited. Ethical approval was obtained from the Jagiellonian University Medical College Ethics Committee (KBET/34/B/2012).

### 2.2 Molecular Genetic Analysis

All samples were screened for mutations in all 18 exons of *LDLR* gene, a fragment of exon 26 of *APOB* to cover p.Arg3527Gln and exon 7 of *PCSK9* to cover p.Asp374Tyr by high resolution melt and direct sequencing of PCR products as described in Supplementary 1. Multiplex ligation-dependent probe amplification to detect gross deletions and insertions in *LDLR* and in silico prediction of pathogenicity of identified variants were performed [8]. The LDL-C gene score was calculated using weighted sums for six LDL-C raising SNPs [5].

### 2.3 Statistical Analysis

The data were not normally distributed and log-transformed data were used for the analysis. One-way ANOVA was used to compare the lipid parameters and gene score between the mutation positive and negative groups (SPSS version 21). p Value < 0.05 was used to denote significance.

## 3 Results

### 3.1 Patient Characteristics

Baseline characteristics of the cohort are shown in Table 1. Mean ± SD maximum total cholesterol (TC) was 9.9 ± 2.6 mmol/L and mean ± SD current LDL-C was 4.8 ± 1.8 mmol/L. Detailed lipid parameters of the individual patients are shown in Supplementary 2.

### 3.2 Mutation Spectrum

Overall we detected a mutation in 70 out of 161 (43.4%) patients. Mutation positive group had a significantly higher TC level (10.5 ± 3.2 vs. 9.5 ± 2.1, p = 0.039) than mutation negative group (Table 1). In 38 patients with a clinical diagnosis of definite FH, we did not find a mutation. The most frequent mutation was in *APOB* (p.Arg3527Gln), found in 13 (8%) patients. No patient carried the *PCSK9* p.Asp374Tyr mutation. Mutations in the *LDLR* gene were identified in 57 patients and accounted for the majority (81.4%) of all the mutations found in this cohort. We identified six different major rearrangements in 12 patients, which accounted for 17.1% of all FH causes in our cohort. Among the intronic variants found, all were previously reported as splice-site-modifying mutations and therefore considered to be pathogenic (www.ucl.ac.uk/ldlr) except c.2390-16G > A which is not near to the splice site; thus, based on prediction tools it was designated as non-pathogenic.

We also identified 13 *LDLR* variants that were considered non-pathogenic. Seven of these variants were present in patients already identified with a pathogenic mutation (Table 2).

### 3.3 Novel Mutations

We found 10 novel mutations in the *LDLR* gene (Table 2). The mutation c.1975_1987 + 16del, is predicted to delete the last four amino acids of exon 13 and the consensus splice site, and is predicted to result in a frame shift. The mutation c.2096delC will also result in a frame shift in exon 14 (p.Pro699Argfs*10) and would be pathogenic. The mutations p.Cys255Tyr and p.Cys329Phe, would cause loss of cysteine in the ligand binding domain of the LDL-receptor and cause aberrant protein folding. The mutation p.Ser849* causes a premature stop codon at position 849 in the cytoplasmic tail of LDL-receptor, known to be important for the localisation of the receptor in coated pits on the cell surface.

**Fig. 1:**
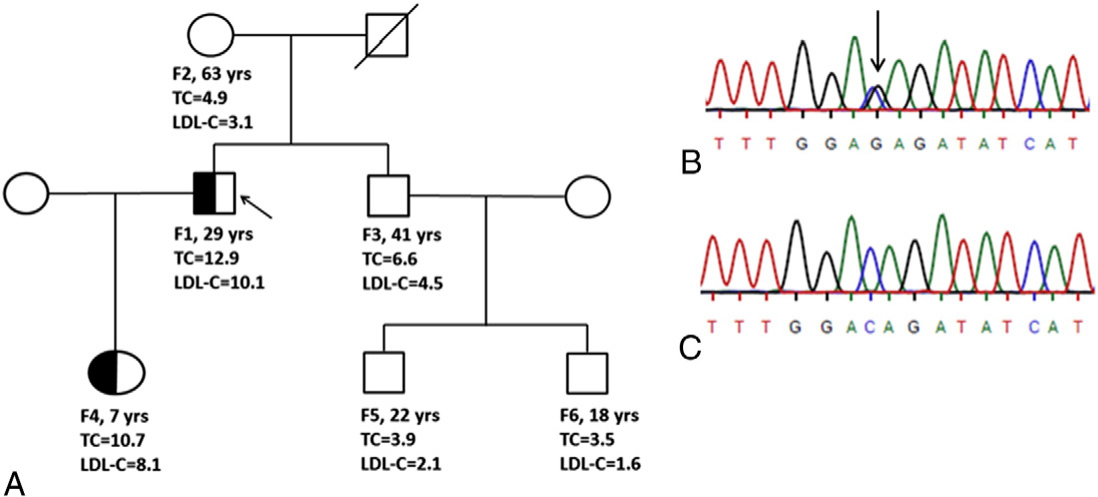
Family co-segregation of the novel c.1862C > G (p.Thr621Arg) *LDLR* mutation. (A) A family pedigree of the index patient (F1) with the novel mutation including age (years), TC level (mmol/L) and LDL-C level (mmol/L). Five members of the family (F2, F3, F4, F5 and F6) were screened and sequenced for the mutation. Only F4 was found to carry the novel variant as the index, which co-segregated with FH phenotype. (B) *LDLR* exon 13 sequencing for the index patient (appropriate base arrowed), (C) Wild type exon 13 sequence.

We predict that the novel mutation (p.The621Arg) would cause aberrant recycling of the LDL-receptor protein to the cell surface and is thus pathogenic. Analysis of the proband’s family members showed that this mutation segregated with the disease. From five family members, the daughter was found to have a raised TC level (10.7 mmol/L) and LDL-C level (8.1 mmol/L) and inherited the p.Thr621Arg mutation. The index father, who had raised serum cholesterol levels, died of myocardial infarction at the age of 46 (Fig. 1).

The other four novel mutations, p.Ala612Ser, p.Asp579Gly, p.Trp483Cys, and p.Val127Asp were also predicted to be pathogenic; however family members of these patients were not available for segregation analysis.

### 3.4 LDL-C Gene Score

**Fig. 2:**
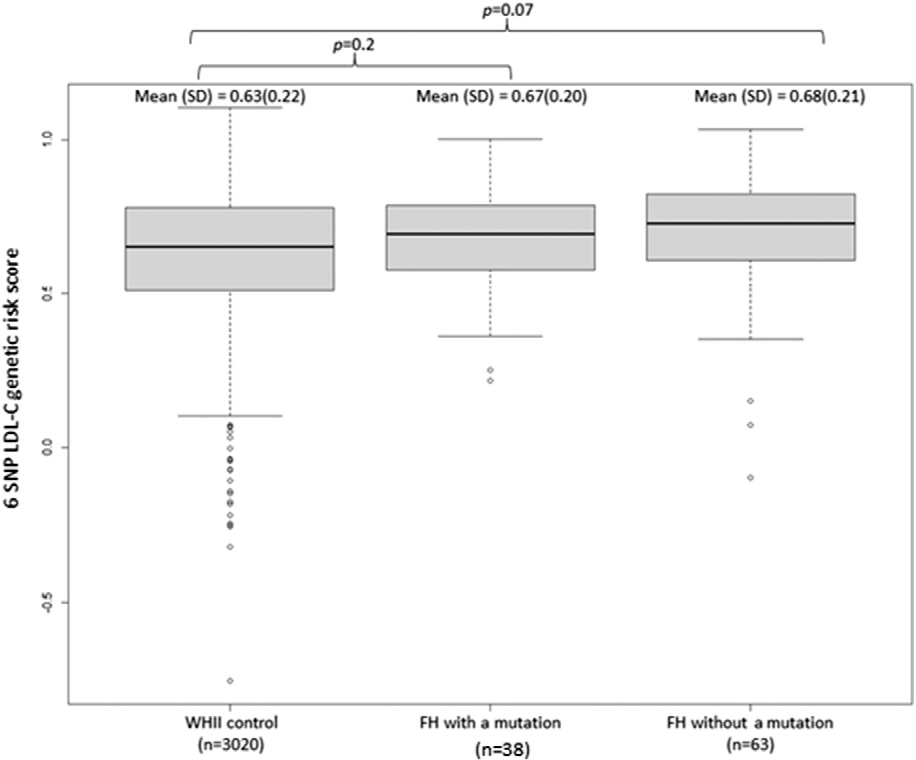
LDL-C genetic risk score analysis based on a 6-SNP score (Futema et al. 2015). Genotypes for 6 LDL-C-associated SNPs were available for 91 out of 101 studied FH patients. For additional nine patients with a one missing *APOE* genotype we assumed that they had the E3E3 isoform (the most common). One more patient had a missing rs6511720 genotype and we assumed that the patient did not have the risk allele for this SNP. The highest mean score (standard deviation (SD)) was observed in individuals with the clinical diagnosis of FH where no mutation detected (0.68 (± 0.21)). Individuals from the control cohort (WHII) had the lowest mean score (SD) (0.63 (± 0.22)), whereas those with a confirmed FH mutation had intermediate score (0.67 (± 0.21)). The differences between the FH patients and the control did not reach a statistical significance.

Genotypes for all six SNPs were obtained for 101 patients. Compared to the control group mean ± SD score (0.63 ± 0.22), the mutation negative patients had the highest LDL-C score (0.68 ± 0.21), followed by the mutation positive patients (0.67 ± 0.21). Following the previously reported trend [5], as expected for a sample of this size, none of these SNP score differences were statistically significant (Fig. 2). Using the control cohort SNP score quartiles, out of the 63 genotyped mutation negative FH patients, 53 (84.1%) had a SNP score above the bottom quartile (> 0.51) and therefore the cause of high LDL-C in these patients is likely to be polygenic.

## 4 Discussion

In this cross-sectional genetic study, we had an overall FH mutation detection rate of 43.4%. This finding is in agreement with previous studies of European populations [9], and similar to that reported in the UK [4], [10]. Approximately 30% of patients with a raised LDL-C level (42.9% of mutation positive and 23.3% of mutation negative) were not on any lipid-lowering medication at the time of recruitment due to their first attendance to lipid clinic for initiation of lipid-lowering drug, statin intolerance or pregnancy. The significantly higher cholesterol levels in monogenic group are likely to be attributed to presence of genetic mutation.

The spectrum of *LDLR* mutations in Europe varies between countries, from Greece with only six mutations responsible for causing FH in 60% of the cases, to Netherlands with the most heterogeneous spectrum [11], [12] and to the UK with over 200 different mutations [13]. We found 39 different FH mutations in a cohort of 161 patients, which suggests a broad spectrum of mutations and high heterogeneity of FH in Poland. The most common *APOB* mutation in European populations p.Arg3527Gln usually accounts for 5–7% of FH patients [14]. We found this mutation in 8% of the patients in south-eastern part of Poland, which is similar to that reported in the northern part of Poland [15], [16]. The frequency of large insertion/deletions was also higher than in a recently reported UK sample (16.7% vs. 10%) [8]. These findings highlight the importance of including the *APOB* gene and large *LDLR* gene rearrangements tests in the mutation screening of Polish people.

We found ten novel pathogenic mutations in the *LDLR* gene based on multiple prediction algorithms and demonstrated co-segregation of the novel mutation p.Thr621Arg with the FH phenotype. We also described the novel mutation of p.Cys329Phe in a previous report [17]. In our study, the mean weighted LDL-C raising SNPs gene score for patients without a mutation was higher than the control group as was shown in previous studies in Europe [5], [18]. In patients where no mutation was found, 84.1% had a gene score in the top three quartiles of the score based on the healthy comparison group, suggesting that they have a polygenic cause for their high cholesterol levels. By contrast, in the remaining 10 mutation-negative patients who were found to have a low SNP score (in the bottom quartile), it is likely that there is a single mutation in a region of the *LDLR*, *APOB* and *PCSK9* genes not examined here, or there might be a mutation in a yet to be discovered gene. Further family studies and use of more comprehensive next generation sequencing methods in these patients may help to distinguish these possibilities.

There are limitations to our study. We had a small number of samples and we only examined the regions of *APOB* and *PCSK9* where the most common FH-causing mutations occur. Also due to lack of consent, we could not perform co-segregation in all patients with novel variants.

The scale of FH under-diagnosis in Poland has been recently highlighted [6], [19] and, as in other European countries, there is an urgent need for a national management plan and an efficient mutation testing strategy in Poland.

## Author Contributions

MSh, MWM, BIW, MTM, MSa, RW, KWL, MF and SHE were involved in analysis, data interpretation and drafting paper. MWM and BIW collected data.

## Conflict of Interest

The authors have no conflict of interest.

## Figures and Tables

**Table 1:** Baseline characteristics of the 161 participants.

Variable	Total	Mutation positive	Mutation negative	p Value
N (%)	N (%)	N (%)
Male	55 (34.2)	26 (37.1)	29 (31.9)	0.48
Tendon xanthomata	92 (57.1)	43 (60.6)	49 (54.4)	0.53
Family history of premature CAD^1^	79 (49.1)	38 (53.5)	41 (45.6)	0.38
Personal history of premature CAD	21 (13.0)	10 (14.1)	11 (12.2)	0.77
On lipid-lowering medication^2^	110 (68.3)	40 (57.1)	70 (76.7)	0.007

	Mean (± SD)	Mean (± SD)	Mean (± SD)	

Age (years)	42 (17.6)	38 (17.9)	46 (16.6)	0.004
Maximum TC (mmol/L)	9.9 (2.6)	10.5 (3.2)	9.5 (2.1)	0.039
LDL-C (mmol/L)^3^	4.8 (1.8)	5.1 (2.0)	4.5 (1.5)	0.046
HDL-C (mmol/L)	1.5 (0.3)	1.4 (0.3)	1.5 (0.3)	0.223
TG (mmol/L)	1.5 (0.8)	1.2 (0.7)	1.7 (0.9)	0.001

1 CAD = coronary artery disease.

2 At the time of study recruitment.

3 Current level.

**Table 2:** LDLR and APOB variants identified in the study.

DNA level	Protein level	N	Exon	Prediction			
PolyPhen	SIFT	Mutation Taster	splice site effect
LDLR gene							
Major rearrangement							
c.-187-?_67 + ?dup	p.(?)	1	promoter-ex1 dup	n/a	n/a	n/a	n/a
c.-187-?_67 + ?del	p.(?)	1	> 30 kb upstream of the promoter-ex1 del	n/a	n/a	n/a	n/a
c.-187-?_190 + ?del	p.(?)	1	promoter-ex2 del	n/a	n/a	n/a	n/a
c.941-?_1060 + ?del	p.Gly314_Glu353del	1	ex7del	n/a	n/a	n/a	No
c.314-?_1186 + ?dup	p.Gly396Ala;Pro106_Val395dup	2	ex4-ex8 dup	n/a	n/a	n/a	No
c.695-?_1586 + ?del	p.Val233Serfs*18	6	ex5-10 del	n/a	n/a	n/a	No

Probably pathogenic							
c.100T > G	p.Cys34Gly	3	2	Probably damaging	Not tolerated	Disease causing	No
c.185C > T	p.Thr62Met	1	2	Probably damaging	Tolerated	Disease causing	No
c.380T > A	p.Val127Asp	1	4^1^	Possibly damaging	Not tolerated	Disease causing	No
c.501C > A	p.Cys167*	1	4	n/a	n/a	n/a	No
c.530C > T	p.Ser177Leu	1	4	Benign	Not tolerated	Disease causing	No
c.654_656delTGG	p.Gly219del	1	4	n/a	n/a	Disease causing	No
c.666C > A	p.Cys222*	1	4	n/a	n/a	n/a	No
c.681C > G	p.Asp227Glu	1	4	Probably damaging	Not tolerated	Disease causing	No
c.764G > A	p.Cys255Tyr	1	5^1^	Probably damaging	Not tolerated	Disease causing	No
c.798T > A	p.Asp266Glu	1	5	Probably damaging	Not tolerated	Disease causing	No
c.986G > T	p.Cys329Phe	4	7^1^	Probably damaging	Not tolerated	Disease causing	No
c.1048C > T	p.Arg350*	1	7	n/a	n/a	n/a	No
c.1085delA	p.Asp362Alafs*8	1	8	n/a	n/a	Disease causing	No
c.1246C > T	p.Arg416Trp	2	9	Probably damaging	Not tolerated	Disease causing	No
c.1449G > T	p.Trp483Cys	1	10^1^	Possibly damaging	Not tolerated	Disease causing	No
c.1567G > A	p.Val523Met	1	10	benign	Not tolerated	Disease causing	No
c.1720C > T	p.Arg574Cys	1	12	Probably damaging	Not tolerated	Disease causing	No
c.1737C > G	p.Asp579Gly	3	12^1^	probably damaging	Not tolerated	Disease causing	No
c.1775G > A	p.Gly592Glu	4	12	probably damaging	Not tolerated	Disease causing	No
c.1834G > T	p.Ala612Ser	2	12^1^	Possibly damaging	Not tolerated	Disease causing	No
c.1862C > G	p.Thr621Arg	1	13^1^	Probably damaging	Not tolerated	Disease causing	No
c.1975_1987 + 16del	p.(?)	1	13^1^	n/a	n/a	n/a	Yes
c.2026G > C	p.Gly676Arg	1	14	Probably damaging	Not tolerated	Disease causing	No
c.2032C > T	p.Gln678*	2	14	n/a	n/a	n/a	No
c.2054C > T	p.Pro685Leu	1	14	Probably damaging	Not tolerated	Disease causing	No
c.2096C > T	p.Pro699Leu	2	14	Probably damaging	Not tolerated	Disease causing	No
c.2096delC	p.Pro699Argfs*10	1	14^1^	n/a	n/a	Disease causing	No
c.2546C > A	p.Ser849*	1	17^1^	n/a	n/a	n/a	No

Intronic pathogenic							
c.313 + 1G > A	p.Leu64_Pro105delinsSer	1	intron 3	n/a	n/a	n/a	Yes
c.1705 + 1G > A		2	intron 11	n/a	n/a	n/a	Yes
c.2140 + 5G > A		2	intron 14	n/a	n/a	n/a	Yes
c.2389 + 5G > A		1	Intron 16	n/a	n/a	n/a	Yes

Non-pathogenic							
c.1171G > A	p.Ala391Thr	3	8	Benign	Tolerated	Polymorphism	No
c.1545C > T	p.Asn515Asn	1	10	n/a	Tolerated	Polymorphism	No
c.1920C > T	p.Asn640Asn	1	13	n/a	n/a	Polymorphism	No
c.1959C > T	p.Val653Val	1	13^1^	n/a	n/a	n/a	No
c.2025C > T	p.Gly675Gly	1	14^1^	n/a	n/a	Disease causing	No
c.2177C > T	p.Thr726Ile	2	15	Benign	Tolerated	Polymorphism	No
c.2231G > A	p.Arg744Gln	1	15	Benign	Tolerated	Polymorphism	No
c.2390-16G > A	Intronic	3	intron 17^1^	n/a	n/a	n/a	No

APOB gene							
c.10580G > A	p.Arg3527Gln	13	APOB ex26	Probably damaging	Not tolerated	n/a	n/a

1 Novel; n/a = not applicable.

## Appendix A Supplementary data

Table S1-1. The primers and PCR conditions used for the *LDLR, APOB* and *PCSK9* genes in HRM. Table S1-2. List of restriction enzymes (NEB (UK) Ltd. Hitchin, Herts, UK) used for RFLP (Restriction Fragment Length Polymorphism). Table S1-3. Global Lipid Genetic Consortium six SNP LDL-C gene score calculations, *APOE* is a major determinant of LDL-C levels and the effect of the *APOE* haplotype was included in the LDL score calculation using effects reported by Bennet et al 2007 [13]. Table S1-4. Baseline characteristics of WHII study participants. Table S2-1. Characteristic of the patients based on the presence or absence of mutation and ranked by Gene Score.
