# Supplementary material for: The genetic spectrum of familial hypercholesterolemia in south-eastern Poland
Source: Metabolism. 2016 Mar;65(3):48–53. doi: 10.1016/j.metabol.2015.10.018 (PMC4766367; doi:10.1016/j.metabol.2015.10.018)
Supplement: Supplementary file 1 — Table S1-1. The primers and PCR conditions used for the LDLR, APOB and PCSK9 genes in HRM. Table S1-2. List of restriction enzymes (NEB (UK) Ltd. Hitchin, Herts, UK) used for RFLP (Restriction Fragment Length Polymorphism). Table S1-3. Global Lipid Genetic Consortium six SNP LDL-C gene score calculations, APOE is a major determinant of LDL-C levels and the effect of the APOE haplotype was included in the LDL score calculation using effects reported by Bennet et al 2007 [13]. Table S1-4. Baseline characteristics of WHII study participants. Table S2-1. Characteristic of the patients based on the presence or absence of mutation and ranked by Gene Score. [file mmc1.doc]

**Supplementary 1**

1. **Methods**
   1. **Molecular Genetic Analysis**

Genomic DNA was extracted from the whole blood samples using standard methods . Ninety nine samples were analysed at the Centre of Cardiovascular Genetics in the UK. These samples were screened for 18 exons of *LDLR* gene, a fragment of exon 26 of *APOB* to cover the area for common mutation p.Arg3527Gln and exon 7 of *PCSK9* to cover p.Asp374Tyr using High Resolution Melt (HRM). Oligonucleotide primers for PCR-HRM were designed to cover intron- exon junctions and up to 40 bp of the intron, and the promoter and coding regions of *LDLR.* Exon 10 of *LDLR* was screened by two overlapping PCR fragments and exon 4 by four overlapping fragments. PCR and subsequent HRM were carried out (as described in Whittall et al ) using the Rotor-Gene 6000 (Qiagen) using AccuMelt HRM SuperMix (Quanta BioSciences, USA) with 25 ng of gDNA and 4 pmol of each primer. The primers and PCR conditions used for the *LDLR*, *APOB* and *PCSK9* genes are given in Supplementary Table S1-1.

Seventy samples were screened for *LDLR* gene mutations at the Jagiellonian University in Poland. In these samples, exon by exon *LDLR* sequence analysis was performed using direct sequencing of PCR products obtained with primers pairs published by Amsellen et al (BigDye 3.1 chemistry; ABI 3500 Genetic Analyser, Applied Biosystems, Life Technologies, Carlsbad, CA). All samples without a mutation in the *LDLR* gene were screened for the *APOB* gene common mutation, p.Arg3527Gln, and sent to Cardiovascular Genetic Lab in the UK for genotyping of *PCSK9* exon 7.

**Table S1-1**: The primers and PCR conditions used for the *LDLR, APOB* and *PCSK9* genes in HRM

| **Gene** | **Fragment** | **primer 5' - 3'** | **Region Covered** |  | **PCR (°C/sec)** |  | **HRM** |
| --- | --- | --- | --- | --- | --- | --- | --- |
|  |  |  |  | Denat | Anneal | Ext | **Temp(°C)** |
| ***LDLR*** | Promoter | CAGCTCTTCACCGGAGACCC | c.-298 - c.-62 | 95/5 | 60/10 | 70/20 | 80-95 |
|  |  | ACCTGCTGTGTCCTAGCTGG |  |  |  |  |  |
|  | Exon 1 | AATCACCCCACTGCAAACTC | c.-139 - c.67+23 | 95/5 | 60/10 | 70/20 | 80-95 |
|  |  | GGGCTCCCTCTCAACCTATT |  |  |  |  |  |
|  | Exon 2 | TTGAGAGACCCTTTCTCCTTTTCC | c.68-10 - c.190+6 | 95/5 | 55/10 | 70/20 | 80-95 |
|  |  | GCATATCATGCCCAAAGGGG |  |  |  |  |  |
|  | Exon 3 | TCAGTGGGTCTTTCCTTTGAG | c.191-28 - c.313+58 | 95/5 | 60/10 | 70/20 | 75-95 |
|  |  | CAGGACCCCGTAGAGACAAA |  |  |  |  |  |
|  | Exon 4 | TGGTGTTGGGAGACTTCACA | c.314-35 - c.519 | 95/5 | 60/10 | 70/20 | 80-95 |
|  |  | CACTCATCCGAGCCATCTTC |  |  |  |  |  |
|  |  | AAGTGCATCTCTCGGCAGTT | c.377 - c.557 | 95/5 | 60/10 | 70/20 | 80-95 |
|  |  | CCCCTTGGAACACGTAAAGA |  |  |  |  |  |
|  |  | AGCTTCCAGTGCAACAGCTC | c.474 - c.679 | 95/5 | 60/10 | 70/20 | 80-95 |
|  |  | CATACCGCAGTTTTCCTCGT |  |  |  |  |  |
|  |  | TGTTCCAAGGGGACAGTAGC | c.586 - c.694+60 | 95/10 | 66/10 | 72/20 | 80-95 |
|  |  | AAATCACTGCATGTCCCACA |  |  |  |  |  |
|  | Exon 5 | AGAAAATCAACACACTCTGTCCTG | c.695-8 - c.817+5 | 95/5 | 60/10 | 70/20 | 80-95 |
|  |  | GGAAAACCAGATGGCCAGCG |  |  |  |  |  |
|  | Exon 6 | TCCTCCTTCCTCTCTCTGGC | c.818-8 - c.940+8 | 95/5 | 60/10 | 70/20 | 80-95 |
|  |  | TCTGCAAGCCGCCTGCACCG |  |  |  |  |  |
|  | Exon 7 | GGCGAAGGGATGGGTAGGGG | c.941-38 - c.1060+36 | 95/5 | 60/10 | 70/20 | 80-95 |
|  |  | GTTGCCATGTCAGGAAGCGC |  |  |  |  |  |
|  | Exon 8 | CTAGCCATTGGGGAAGAGCC | c.1061-31 - c.1186+30 | 95/5 | 60/10 | 70/20 | 80-95 |
|  |  | TGCCTGCAAGGGGTGAGGC |  |  |  |  |  |
|  | Exon 9 | TCCATCGACGGGTCCCCTCTGACCC | c.1187-26 - c.1358+25 | 95/5 | 60/10 | 70/20 | 80-95 |
|  |  | AGCCCTCATCTCACCTGCGGGCCAA |  |  |  |  |  |
|  | Exon 10 | AGATGAGGGCTCCTGGTGCGATGCC | c.1359-26 - c.1490 | 95/5 | 60/10 | 70/20 | 80-95 |
|  |  | GCCCTTGGTATCCGCAACAGAGACA |  |  |  |  |  |
|  |  | GATCCACAGCAACATCTACTGGACC | c.1475 - c.1586+5 | 95/5 | 60/10 | 70/20 | 80-95 |
|  |  | AGCCCTCAGCGTCGTGGATA |  |  |  |  |  |
|  | Exon 11 | TCCTCCCCCGCCCTCCAGCC | c.1587-28 - c.1705+7 | 95/5 | 60/10 | 70/20 | 75-90 |
|  |  | GCTGGGACGGCTGTCCTGCG |  |  |  |  |  |
|  | Exon 12 | GCACGTGACCTCTCCTTATCCACTT | c.1706-10 - c.1845+10 | 95/5 | 56/20 | 70/30 | 80-90 |
|  |  | CACCTAAGTGCTTCGATCTCGTACG |  |  |  |  |  |
|  | Exon 13 | AGAGGGTGGCCTGTGTCTC | c.1846-47 - c.1987+29 | 95/5 | 58/10 | 70/20 | 78-90 |
|  |  | TCCACAAGGAGGTTTCAAGG |  |  |  |  |  |
|  | Exon 14 | CTGATGATCTCGTTCCTGCCC | c.1988-23 - c.2140+46 | 95/5 | 60/10 | 70/20 | 80-95 |
|  |  | GCAGAGAGAGGCTCAGGAGG |  |  |  |  |  |
|  | Exon 15 | GGCACGTGGCACTCAGAAGAC | c.2141-18 - c.2311+25 | 95/5 | 60/10 | 70/20 | 80-95 |
|  |  | ACCCGTCTCTGGGTGAAGAGG |  |  |  |  |  |
|  | Exon 16 | CCTTCCTTTAGACCTGGGCC | c.2312-23 - c.2389+32 | 95/5 | 60/10 | 70/20 | 80-95 |
|  |  | CATAGCGGGAGGCTGTGACC |  |  |  |  |  |
|  | Exon 17 | GGGTCTCTGGTCTCGGGCGC | c.2390-33 - c.2547+10 | 95/5 | 60/10 | 70/20 | 80-95 |
|  |  | GGCTCTGGCTTTCTAGAGAGGG |  |  |  |  |  |
|  | Exon 18 | GCCTGTTTCCTGAGTGCTGG | c.2548-35 - c.2607 | 95/5 | 60/10 | 70/20 | 80-95 |
|  |  | TCTCAGGAAGGGTTCTGGGC |  |  |  |  |  |
| ***PCSK9*** | Exon 7 | CCCTCTCTTGGGCTCCTTTCT | c.997-27 - c.1180+29 | 95/10 | 60/10 | 70/20 | 78-94 |
|  |  | AAAGGGGCTGTTAGCATCACG |  |  |  |  |  |
| ***APOB*** | Exon 26 | TGTCAAGGGTTCGGTTCTTT | c.10516 - c.10745 | 95/5 | 60/10 | 70/20 | 80-95 |
|  |  | GGGTGGCTTTGCTTGTATGT |  |  |  |  |  |

- 1. **Restriction Fragment Length Polymorphism (RFLP)**

Samples were genotyped for common polymorphisms in the *LDLR* by RFLP and their genotypes compared with the HRM result as described previously . Those with shifts due to polymorphism on the HRM were not examined further. However, samples without the polymorphism but with an HRM shift were sequenced to find the cause of the melt temperature shift. The PCR products were used for digestion with the appropriate restriction enzyme (NEB (UK) Ltd. Hitchin, Herts, UK) using 5 μl of HRM-PCR product and 3U of appropriate enzyme (Supplementary Table S1-2) in a total volume of 15 μl and run on a 1.5% agarose gel in 1xTBE buffer.

**Table S1-2**: List of restriction enzymes (NEB (UK) Ltd. Hitchin, Herts, UK) used for RFLP (Restriction Fragment Length Polymorphism)

| ***LDLR* exon** | **Enzyme** | **Polymorphism** |
| --- | --- | --- |
| **ex 2** | Hhal | rs2228671 |
| **ex 7** | SmaI | rs12710260 |
| **ex 8** | StuI | rs11669576 |
| **ex 10** | BsmA1 | rs5930 |
| **ex 11** | AciI | rs5929 |
| **ex 12** | BSMAI & HincII | rs1799898 & rs688 |
| **ex 13** | AvaII | rs5925 |
| **ex 14** | MSII | rs72658867 |
| **ex 15** | MspI | rs5927 |

- 1. **Sequence Analysis**

The HRM-PCR products that showed a shift in the melt profile and melt temperature, were Sanger sequenced. The DNA purification was performed using Illustra GFX PCR DNA and Gel Band Purification Kit (from GE Healthcare). The same primers used for HRM were used for sequencing, which was performed by Source BioSciences, LifeSciences.

- 1. **Segregation analysis and mutation screening**

To assess the family co-segregation of the novel *LDLR* variant p.Thr621Arg with the FH phenotype, five family members of the mutation carrier (3 first-degree and 2 second-degree relatives) were screened in Poland and their DNA samples were sent to the UK and sequenced for the same mutation, as described above.

- 1. **Multiplex Ligation Dependent Probe Amplification (MLPA)**

To detect rearrangements within the coding sequence of *LDLR*, the multiplex ligation-dependent probe amplification (MLPA) was performed according to the manufacturer’s protocol on all samples (MRC-Holland, Amsterdam, the Netherlands).

- 1. **In silico analysis**

To predict the pathogenicity of the novel *LDLR* variants, *in silico* mutation prediction tools, including Polymorphism Phenotyping version2 (PolyPhen-2), Scale-invariant feature transform (SIFT), Berkeley Drosophila Genome Project - Splice Site Prediction (BDGP) and Mutation Taster were used in all samples. The data from Exome Variant Server (<http://evs.gs.washington.edu/EVS/>) and UCL website ([www.ucl.ac.uk/fh](http://www.ucl.ac.uk/fh)) were also used to check if any of the mutations were novel. Mutation nucleotide numbers were designated using the *LDLR* sequence reported in ([www.ucl.ac.uk/fh](http://www.ucl.ac.uk/fh)) . Mutations were designated according to recommendations from Human Genome Variation Society ([www.hgvs.org](http://www.hgvs.org/)). Information from the novel mutations was added to the UCL *LDLR* database ([www.ucl.ac.uk/fh](http://www.ucl.ac.uk/fh)).

- 1. **LDL-C gene score calculations for polygenic hypercholesterolemia**

To assess the polygenic cause of hypercholesterolemia, patients were genotyped for six LDL-C-raising SNPs based on recent findings . KASPar PCR technique (Kbiosciences, UK Hoddesdon, Herts, UK) or TaqMan assays (Life Technologies, Carlsbad, California, US) and genotype calling for all assays was carried out using an automated system, the results of which were checked manually by study personnel using SNPviewer software. One SNP (rs4299376) could not be genotyped and a proxy was used instead (rs6544731). The LDL-C gene score was calculated using weighted sums for six SNPs of the highest effect (see Supplementary Table S1-3). A group of 3,020 healthy volunteers (participants of the UK Whitehall II (WHII) study ) was used for comparison (baseline characteristics of WHII are shown in Supplementary Table S1-4). Patients were grouped into quartiles of the gene score based on the WHII population. It has been estimated using probability calculations that patients in the top three quartiles of the score have a greater than 98% probability of having a polygenic cause of their hypercholesterolemia.

**Table S1-3**: Global Lipid Genetic Consortium six SNP LDL-C gene score calculations,

*APOE* is a major determinant of LDL-C levels and the effect of the *APOE* haplotype was included in the LDL score calculation using effects reported by Bennet et al 2007 .

|  | **Chromosome** | **Gene** | **Minor allele** | **Common allele** | **Weight for score calculation** |
| --- | --- | --- | --- | --- | --- |
| rs629301 | 1 | *CELSR2* | G | T¹ | 0.15 |
| rs1367117 | 2 | *APOB* | A¹ | G | 0.1 |
| rs4299376 | 2 | *ABCG8* | G¹ | T | 0.071 |
| rs6511720 | 19 | *LDLR* | T | G¹ | 0.18 |
| rs429358 | 19 | *APOE* | C | T | . |
| rs7412 | 19 | *APOE* | T | C | . |
| ε2ε2 | 19 | *APOE* | . | . | -0.9 |
| ε2ε3 | 19 | *APOE* | . | . | -0.4 |
| ε2ε4 | 19 | *APOE* | . | . | -0.2 |
| ε3ε3 | 19 | *APOE* | . | . | 0 |
| ε3ε4 | 19 | *APOE* | . | . | 0.1 |
| ε4ε4 | 19 | *APOE* | . | . | 0.2 |

¹Risk alleles (LDL-C raising)

**Table S1-4**: Baseline characteristics of WHII study participants

|  | **WHII (n=3020)** |
| --- | --- |
| % male | 76% (2308/3020) |
| Age (years) | 49.0 ± 6.0 |
| Pre-treatment TC (mmol/l) | 6.4 ± 1.1 |
| Pre-treatment LDL-C (mmol/l) | 4.4 ± 1.0 |

- 1. **Lipid concentration measurements**

Lipid concentrations were measured by Roche enzymatic method .

**Supplementary 2**

**Table S2-1: Characteristic of the patients based on the presence or absence of mutation and ranked by Gene Score**

| ID no. | sex | Age | TC¹ | LDL¹ | HDL¹ | TG¹ | Treatment² | Max TC | Total 6-SNPs |
| --- | --- | --- | --- | --- | --- | --- | --- | --- | --- |
|  |  |  | (mmol/l) | (mmol/l | mmol/l) | (mmol/l) |  | (mmol/l) | score⁵ |
| mut pos |  |  |  |  |  |  |  |  |  |
| 1 | F | 52 | 10.5 | 8.0 | 1.5 | 2.0 | Atorva | - | 0.22 |
| 2 | M | 40 | 9.8 | 7.9 | 1.1 | 1.6 | - | 10.6 | 0.25 |
| 3 | F | 62 | 5.2 | 2.9 | 1.9 | 0.9 | Rosuva | 10.3 | 0.36 |
| 4 | F | 56 | 6.7 | 4.7 | 1.2 | 1.9 | Rosuva, Ez | 15.5 | 0.36³ |
| 5 | F | 50 | 5.6 | 3.8 | 1.5 | 0.7 | Atorva | 9.1 | 0.40 |
| 6 | F | 30 | 6.9 | 4.9 | 1.5 | 1.2 | - | 8.0 | 0.43 |
| 7 | F | 14 | 7.5 | 5.6 | 1.3 | 1.2 | - | 9.2 | 0.48 |
| 8 | M | 28 | 8.8 | 7.0 | 1.3 | 1.2 | - | 8.8 | 0.51 |
| 9 | F | 63 | 7.1 | 4.9 | 1.8 | 0.8 | - | 10.3 | 0.51³ |
| 10 | F | 5 | 6.8 | 4.7 | 1.7 | 0.8 | - | 7.8 | 0.58 |
| 11 | F | 56 | 7.6 | 5.9 | 2.0 | 1.7 | - | 12.0 | 0.58 |
| 12 | M | 11 | 4.5 | 3.1 | 0.9 | 0.8 | Vasosan | 7.9 | 0.60 |
| 13 | F | 16 | 4.4 | 2.7 | 1.3 | 0.7 | Atorva | 8.3 | 0.61 |
| 14 | M | 58 | 5.6 | 3.0 | 1.2 | 3.1 | Atorva | 10.0 | 0.61 |
| 15 | F | 44 | 10.6 | 8.7 | 1.4 | 1.2 | - | 11.2 | 0.65 |
| 16 | M | 27 | 11.2 | 9.5 | 1.3 | 0.9 | - | 11.9 | 0.65 |
| 17 | M | 43 | 11.7 | 9.4 | 1.9 | 0.9 | - | - | 0.65 |
| 18 | M | 64 | 5.7 | 3.7 | 1.2 | 1.7 | Atorva, Ez | 9.7 | 0.66 |
| 19 | F | 62 | 5.2 | 3.2 | 1.6 | 0.8 | Atorva, Ez | 14.0 | 0.68 |
| 20 | F | 28 | 6.3 | 4.3 | 1.6 | 0.9 | Simva | 9.5 | 0.71 |
| 21 | M | 72 | 8.7 | 5.3 | 1.2 | 4.6 | Rosuva, Ez | 12.0 | 0.72 |
| 22 | M | 29 | 12.9 | 10.1 | 1.2 | 3.4 | Simva | 14.5 | 0.72 |
| 23 | F | 64 | 6.1 | 4.0 | 1.4 | 1.6 | Simva | 9.5 | 0.73 |
| 24 | F | 28 | 4.4 | 2.1 | 1.9 | 0.8 | Atorva | 8.2 | 0.73 |
| 25 | M | 50 | 13.2 | 10.6 | 1.6 | 2.2 | Rosuva | 14.3 | 0.75 |
| 26 | M | 19 | 7.4 | 5.9 | 1.2 | 0.7 | - | 7.3 | 0.76 |
| 27 | M | 47 | 8.0 | 6.0 | 1.3 | 1.4 | - | 12.0 | 0.78 |
| 28 | M | 36 | 5.0 | 3.1 | 1.4 | 1.0 | Rosuva | 10.4 | 0.78 |
| 29 | F | 26 | 8.9 | 7.2 | 1.2 | 1.0 | - | - | 0.79 |
| 30 | F | 65 | 7.1 | 4.7 | 1.5 | 1.0 | Simva | 10.7 | 0.83 |
| 31 | M | 54 | 6.4 | 4.7 | 1.0 | 1.6 | Rosuva, Ez | - | 0.83 |
| 32 | F | 50 | 6.1 | 3.4 | 1.4 | 2.9 | Simva | 11.2 | 0.83 |
| 33 | F | 55 | 5.0 | 2.6 | 1.9 | 1.1 | Rosuva | 9.0 | 0.83 |
| 34 | F | 38 | 7.8 | 4.9 | 2.3 | 1.2 | Atorva | 10.0 | 0.90 |
| 35 | F | 33 | 7.3 | 5.0 | 2.0 | 0.7 | - | 8.0 | 0.93 |
| 36 | F | 28 | 8.2 | 5.7 | 2.1 | 0.9 | - | 8.4 | 0.95 |
| 37 | F | 50 | 5.3 | 3.6 | 1.2 | 1.1 | Rosuva | 10.5 | 1.00 |
| 38 | F | 28 | 4.8 | 2.9 | 1.7 | 0.5 | Rosuva | 8.2 | 1.00 |
| 39 | M | 34 | 8.1 | 5.9 | 1.2 | 2.1 | Atorva | 11.0 | - |
| 40 | F | 35 | 6.9 | 5.0 | 1.5 | 0.8 | Atorva | 14.0 | - |
| 41 | M | 19 | 7.2 | 5.7 | 1.2 | 0.6 | - | - | - |
| 42 | M | 45 | 7.8 | 5.8 | 1.4 | 1.4 | Atorva | 7.0 | - |
| 43 | F | 73 | 6.2 | 4.1 | 1.4 | 1.6 | Atorva | 15.0 | - |
| 44 | F | 34 | 5.7 | 3.6 | 1.6 | 1.1 | - | 7.1 | - |
| 45 | M | 48 | 7.1 | 4.9 | 1.8 | 0.8 | Atorva | 11.0 | - |
| 46 | M | 67 | 4.8 | 2.8 | 1.7 | 0.6 | Atorva | 9.0 | - |
| 47 | F | 42 | 6.7 | 4.3 | 2.0 | 1.0 | Atorva | 10.5 | - |
| 48 | F | 56 | 7.8 | 5.8 | 1.2 | 1.9 | Atorva, Ez | 15.4 | - |
| 49 | M | 40 | 5.9 | 4.3 | 1.0 | 1.4 | Atorva | 8.1 | - |
| 50 | F | 40 | 9.0 | 7.3 | 1.3 | 0.8 | Atorva | - | - |
| 51 | F | 44 | 8.9 | 6.5 | 2.1 | 0.8 | Lipanthyl | 10.7 | - |
| 52 | F | 55 | 7.4 | 5.0 | 1.9 | 1.0 | Atorva | 11.5 | - |
| 53 | F | 31 | 9.8 | 8.0 | 1.4 | 1.0 | - | 12.9 | - |
| 54 | F | 52 | 7.0 | 5.2 | 1.4 | 1.1 | Atorva | 16.6 | - |
| 55 | M | 29 | 6.5 | 4.5 | 1.5 | 1.0 | Simva | 8.3 | - |
| 56 | F | 40 | 8.3 | 6.7 | 1.2 | 0.8 | - | 9.9 | - |
| 57 | F | 40 | 7.3 | 5.4 | 1.6 | 0.8 | - | 10.0 | - |
| 58 | F | 24 | 6.4 | 4.2 | 1.7 | 1.0 | - | 7.5 | - |
| 59 | F | 53 | 5.1 | 3.5 | 1.2 | 0.9 | Simva | 9.6 | - |
| 60 | F | 56 | 6.6 | 4.4 | 1.7 | 1.0 | Atorva | 9.0 | - |
| 61 | F | 35 | 8.5 | 6.7 | 1.4 | 1.0 | - | - | - |
| 62 | F | 16 | 5.4 | 3.7 | 1.2 | 1.1 | - | 6.6 | - |
| 63 | M | 4 | 4.4 | 2.0 | 2.1 | 0.6 | Atorva | 9.6 | - |
| 64 | M | 13 | 6.7 | 4.8 | 0.8 | 3.3 | - | 6.6 | - |
| 65 | M | 9 | 8.6 | 7.2. | 1.0 | 1.0 | - | 27.0 | - |
| 66 | F | 8 | 8.0 | 5.5 | 1.2 | 0.8 | - | - | - |
| 67 | F | 8 | 8.4 | 6.3 | 1.6 | 1.1 | - | - | - |
| 68 | M | 15 | 6.0 | 4.3 | 1.4 | 0.6 | - | 8.2 | - |
| 69 | M | 15 | 8.4 | 6.1 | 1.6 | 1.0 | - | 9.0 | - |
| 70 | F | 21 | 10.6 | 8.7 | 1.6 | 0.7 | - | - | - |
|  |  |  |  |  |  |  |  |  |  |
| mut neg |  |  |  |  |  |  |  |  |  |
| 71 | F | 65 | 5.9 | 2.8 | 1.4 | 3.7 | Rosuva | 14.6 | -0.10 |
| 72 | F | 67 | 9.2 | 5.5 | 2.1 | 3.3 | - | 10.0 | 0.07 |
| 73 | F | 58 | 5.5 | 3.3 | 1.7 | 1.3 | Simva | 11.8 | 0.15 |
| 74 | F | 59 | 9.5 | 5.7 | 1.3 | 5.2 | - | 9.2 | 0.35 |
| 75 | F | 37 | 6.7 | 4.4 | 1.1 | 2.6 | Rosuva | 15.5 | 0.43 |
| 76 | F | 60 | 6.8 | 4.6 | 1.4 | 1.7 | Atorva | 12.0 | 0.47 |
| 77 | F | 60 | 5.0 | 2.3 | 1.9 | 1.7 | Atorva | 7.8 | 0.47 |
| 78 | F | 59 | 5.9 | 3.4 | 1.6 | 1.9 | Simva | 9.6 | 0.47 |
| 79 | F | 50 | 9.9 | 6.8 | 1.9 | 2.8 | Simva | 10.2 | 0.51 |
| 80 | F | 22 | 10.1 | 8.3 | 1.1 | 1.6 | - | - | 0.51 |
| 81 | M | 33 | 6.4 | 4.2 | 1.6 | 1.4 | - | 7.8 | 0.51 |
| 82 | F | 58 | 7.1 | 4.7 | 1.5 | 2.0 | Rosuva | 8.2 | 0.51³ |
| 83 | M | 66 | 8.1 | 5.4 | 1.5 | 2.5 | Rosuva | 8.1 | 0.53 |
| 84 | F | 62 | 8.3 | 6.0 | 1.1 | 2.5 | atorva | - | 0.54 |
| 85 | M | 53 | 5.7 | 3.2 | 1.0 | 3.3 | Lovastatin | 8.5 | 0.55 |
| 86 | F | 55 | 8.9 | 6.2 | 1.3 | 3.2 | Simva | 9.1 | 0.60 |
| 87 | M | 7 | 6.0 | 4.2 | 1.2 | 1.4 | - | - | 0.61 |
| 88 | F | 50 | 8.8 | 6.5 | 1.6 | 1.4 | - | 9.5 | 0.65 |
| 89 | F | 29 | 7.1 | 5.0 | 1.6 | 1.2 | Rosuva | 11.0 | 0.65 |
| 90 | F | 60 | 7.3 | 4.8 | 1.8 | 1.5 | Fluva | 9.2 | 0.65 |
| 91 | F | 41 | 7.8 | 5.7 | 1.7 | 0.8 | - | - | 0.65 |
| 92 | M | 28 | 8.3 | 6.1 | 1.6 | 1.3 | - | 11.8 | 0.65 |
| 93 | M | 32 | 5.1 | 3.1 | 1.3 | 1.6 | Simva | - | 0.66 |
| 94 | F | 56 | 10.0 | 7.4 | 1.7 | 2.1 | Rosuva | 9.0 | 0.66 |
| 95 | F | 56 | 8.4 | 6.2 | 1.2 | 2.3 | - | 8.8 | 0.66 |
| 96 | F | 53 | 7.8 | 5.0 | 1.4 | 3.0 | - | 9.4 | 0.66 |
| 97 | F | 8 | 5.6 | 4.0 | 0.9 | 1.4 | vasosan | 6.8 | 0.68 |
| 98 | F | 54 | 5.6 | 3.4 | 1.6 | 1.1 | Simva | 5.6 | 0.71 |
| 99 | F | 24 | 10.1 | 8.2 | 1.6 | 0.7 | simva | 16.8 | 0.72 |
| 100 | M | 48 | 8.6 | 6.3 | 1.5 | 1.7 | atorva | - | 0.72 |
| 101 | M | 27 | 7.5 | 4.4 | 0.9 | 4.8 | - | 15.5 | 0.73 |
| 102 | M | 37 | 5.6 | 3.1 | 1.7 | 1.8 | Rosuva | 8.9 | 0.73 |
| 103 | M | 38 | 4.6 | 2.4 | 1.9 | 0.7 | Atorva | 7.0 | 0.73 |
| 104 | F | 46 | 5.8 | 3.7 | 1.7 | 0.8 | Simva | 9.1 | 0.73³ |
| 105 | F | 58 | 7.8 | 5.4 | 1.5 | 2.0 | Fluva | 10.4 | 0.75 |
| 106 | F | 37 | 8.3 | 6.1 | 1.8 | 0.9 | - | 9.9 | 0.75 |
| 107 | F | 52 | 5.3 | 2.9 | 1.9 | 1.1 | Rosuva | 11.1 | 0.75 |
| 108 | F | 55 | 8.1 | 5.3 | 1.4 | 3.0 | Simva | 10.2 | 0.76 |
| 109 | M | 56 | 7.4 | 4.3 | 2.8 | 0.7 | Simva | 12.4 | 0.76 |
| 110 | F | 22 | 5.1 | 2.2 | 1.9 | 2.1 | Simva | 8.2 | 0.76 |
| 111 | M | 64 | 8.5 | 5.7 | 2.2 | 1.2 | - | 8.1 | 0.76 |
| 112 | M | 48 | 7.0 | 4.9 | 0.8 | 3.0 | lipanthyl | 11.2 | 0.76³ |
| 113 | F | 8 | 6.5 | 4.4 | 1.8 | 0.8 | - | 6.4 | 0.76³ |
| 114 | M | 8 | 6.6 | 4.9 | 1.3 | 1.0 | - | 7.1 | 0.76³ |
| 115 | M | 6 | 4.0 | 2.3 | 1.3 | 0.9 | vasosan | 6.2 | 0.76 |
| 116 | M | 51 | 6.4 | 3.8 | 1.6 | 2.1 | Simva | 10.0 | 0.80 |
| 117 | F | 67 | 4.3 | 2.0 | 1.9 | 0.8 | Rosuva | 8.4 | 0.82 |
| 118 | F | 52 | 7.4 | 4.7 | 2.0 | 1.5 | - | 9.5 | 0.83 |
| 119 | F | 60 | 5.7 | 3.1 | 1.3 | 2.9 | Simva | - | 0.83 |
| 120 | F | 57 | 5.6 | 3.5 | 1.3 | 1.8 | Simva | 9.8 | 0.83³ |
| 121 | M | 55 | 5.5 | 3.5 | 1.3 | 1.5 | Atorva | 9.1 | 0.83 |
| 122 | M | 44 | 9.7 | 7.0 | 1.7 | 2.1 | Rosu | 10.0 | 0.83 |
| 123 | M | 43 | 8.1 | 5.6 | 1.5 | 2.3 | Rosuva | 9.0 | 0.83 |
| 124 | M | 38 | 6.9 | 4.8 | 1.6 | 1.0 | Simva | 9.7 | 0.85 |
| 125 | F | 16 | 6.6 | 4.1 | 1.8 | 1.6 | - | 5.5 | 0.86 |
| 126 | M | 38 | 7.2 | 5.1 | 1.5 | 1.4 | - | 10.0 | 0.90 |
| 127 | F | 54 | 6.2 | 3.2 | 2.6 | 0.7 | Simva | 10.2 | 0.93 |
| 128 | F | 29 | 8.7 | 6.9 | 1.4 | 0.8 | - | 7.9 | 0.93 |
| 129 | M | 31 | 10.8 | 9.1 | 1.2 | 1.4 | fibrate | 9.2 | 0.93³ |
| 130 | F | 58 | 9.3 | 6.2 | 2.4 | 1.3 | Rosuva, Ez | 9.8 | 0.96 |
| 131 | M | 47 | 7.6 | 5.5 | 1.7 | 0.9 | Fluva | 7.8 | 1.01 |
| 132 | M | 48 | 6.8 | 4.3 | 1.2 | 2.5 | Simva | 8.4 | 1.03 |
| 133 | F | 57 | 6.2 | 3.6 | 2.0 | 1.1 | Atorva | 10.8 | - |
| 134 | F | 49 | 11.5 | 9.6 | 1.1 | 1.7 | Simva | 15.5 | - |
| 135 | F | 55 | 6.8 | 4.5 | 1.7 | 1.3 | Atorva | 10.0 | - |
| 136 | F | 57 | 6.3 | 3.7 | 1.4 | 2.6 | Atorva | 9.3 | - |
| 137 | F | 21 | 8.3 | 6.3 | 1.5 | 1.1 | - | 8.7 | - |
| 138 | F | 54 | 4.4 | 2.3 | 1.6 | 1.3 | Atorva | 8.2 | - |
| 139 | F | 39 | 6.8 | 4.8 | 1.5 | 1.3 | Atorva | 11.0 | - |
| 140 | F | 58 | 5.6 | 3.9 | 1.1 | 1.4 | Fluva | 9.0 | - |
| 141 | F | 68 | 6.2 | 3.5 | 2.5 | 0.4 | Simva | 9.6 | - |
| 142 | F | 55 | 5.3 | 3.2 | 1.5 | 1.1 | Atorva | 10.8 | - |
| 143 | F | 65 | 5.3 | 3.1 | 1.5 | 1.5 | Atorva | 9.2 | - |
| 144 | F | 59 | 4.6 | 2.4 | 1.7 | 0.8 | Atorva | 8.6 | - |
| 145 | M | 56 | 7.5 | 5.9 | 1.2 | 1.1 | Atorva | 9.8 | - |
| 146 | M | 30 | 3.9 | 2.7 | 0.9 | 0.8 | Atorva, Ez | 9.3 | - |
| 147 | F | 62 | 7.6 | 5.2 | 1.6 | 1.8 | Ezetrol | 9.3 | - |
| 148 | F | 58 | 7.2 | 5.0 | 1.8 | 0.9 | vasosan | 11.2 | - |
| 149 | F | 42 | 5.7 | 3.5 | 1.6 | 1.1 | Atorva | 9.2 | - |
| 150 | F | 62 | 5.9 | 3.3 | 1.3 | 2.9 | Ez | 8.5 | - |
| 151 | M | 42 | 6.8 | 4.1 | 1.1 | 3.5 | - | 7.1 | - |
| 152 | F | 59 | 4.9 | 2.7 | 1.6 | 1.4 | Atorva | 7.1 | - |
| 153 | F | 34 | 8.4 | 6.2 | 1.5 | 1.6 | Simva | 11.7 | - |
| 154 | F | 52 | 7.9 | 5.2 | 2.2 | 1.1 | Simva | 9.8 | - |
| 155 | F | 59 | 4.9 | 2.0 | 1.7 | 2.7 | Simva | 9.9 | - |
| 156 | M | 56 | 5.0 | 3.2 | 1.2 | 1.2 | Atorva | 10.7 | - |
| 157 | F | 17 | 6.2 | 4.5 | 1.3 | 0.9 | Atorva | 7.7 | - |
| 158 | M | 15 | 5.0 | 3.5 | 0.9 | 1.3 | Vasosan | 7.2 | - |
| 159 | F | 58 | 7.2 | 5.0 | 1.8 | 0.9 | Vasosan | 11.2 | - |
| 160 | F | 27 | 5.6 | 3.6 | 1.5 | 1.0 | Rosuva | 7.0 | - |
| 161 | F | 84 | 5.3 | 3.1 | 1.5 | 1.4 | Atorva | - | - |
|  |  |  |  |  |  |  |  |  |  |

¹Current level; ²Atova=Atorvastatin, Fluva=Fluvastatin, Ez=Ezetimibe, Rosuva= Rosuvastatin and Simva=Simvastatin; ³Missing *APOE* genotype; we assumed that the patient is E3/E3; Missing rs6511720 genotype; we assumed that the patient does not carry the risk allele; ⁵DNA samples for LDL-C gene scoring were only available in 101 patients.

**Supplementary References**

1. Miller SA, Dykes DD and Polesky HF A simple salting out procedure for extracting DNA from human nucleated cells. Nucleic Acids Res 1988; 16(3): 1215.

2. Whittall RA, Scartezini M, Li K, Hubbart C, Reiner Z, Abraha A et al. Development of a high-resolution melting method for mutation detection in familial hypercholesterolaemia patients. Ann Clin Biochem 2010; 47(Pt 1): 44-55.

3. Amsellem S, Briffaut D, Carrie A, Rabes JP, Girardet JP, Fredenrich A et al. Intronic mutations outside of Alu-repeat-rich domains of the LDL receptor gene are a cause of familial hypercholesterolemia. Hum Genet 2002; 111(6): 501-10.

4. Adzhubei IA, Schmidt S, Peshkin L, Ramensky VE, Gerasimova A, Bork P et al. A method and server for predicting damaging missense mutations. Nat Methods 2010; 7(4): 248-9.

5. Ng PC and Henikoff S Predicting deleterious amino acid substitutions. Genome Res 2001; 11(5): 863-74.

6. Hoskins RA, Carlson JW, Kennedy C, Acevedo D, Evans-Holm M, Frise E et al. Sequence finishing and mapping of Drosophila melanogaster heterochromatin. Science 2007; 316(5831): 1625-8.

7. Schwarz JM, Rodelsperger C, Schuelke M and Seelow D MutationTaster evaluates disease-causing potential of sequence alterations. Nat Methods 2010; 7(8): 575-6.

8. Fokkema IF, den Dunnen JT and Taschner PE LOVD: easy creation of a locus-specific sequence variation database using an "LSDB-in-a-box" approach. Hum Mutat 2005; 26(2): 63-8.

9. Teslovich TM, Musunuru K, Smith AV, Edmondson AC, Stylianou IM, Koseki M et al. Biological, clinical and population relevance of 95 loci for blood lipids. Nature 2010; 466(7307): 707-13.

10. Talmud PJ, Shah S, Whittall R, Futema M, Howard P, Cooper JA et al. Use of low-density lipoprotein cholesterol gene score to distinguish patients with polygenic and monogenic familial hypercholesterolaemia: a case-control study. Lancet 2013; 381(9874): 1293-301.

11. Futema M, Shah S, Cooper JA, Li K, Whittall RA, Sharifi M et al. Refinement of Variant Selection for the LDL Cholesterol Genetic Risk Score in the Diagnosis of the Polygenic Form of Clinical Familial Hypercholesterolemia and Replication in Samples from 6 Countries. Clin Chem 2015; 61(1): 231-8.

12. Marmot MG, Smith GD, Stansfeld S, Patel C, North F, Head J et al. Health inequalities among British civil servants: the Whitehall II study. Lancet 1991; 337(8754): 1387-93.

13. Bennet AM, Di Angelantonio E, Ye Z, Wensley F, Dahlin A, Ahlbom A et al. Association of apolipoprotein E genotypes with lipid levels and coronary risk. JAMA 2007; 298(11): 1300-11.

14. Ordonez-Llanos J, Wagner AM, Bonet-Marques R, Sanchez-Quesada JL, Blanco-Vaca F and Gonzalez-Sastre F Which cholesterol are we measuring with the Roche direct, homogeneous LDL-C Plus assay? Clin Chem 2001; 47(1): 124-6.

15. Neil A, Cooper J, Betteridge J, Capps N, McDowell I, Durrington P et al. Reductions in all-cause, cancer, and coronary mortality in statin-treated patients with heterozygous familial hypercholesterolaemia: a prospective registry study. Eur Heart J 2008; 29(21): 2625-33.
